# Differences in the pathogenetic characteristics of prostate cancer in the transitional and peripheral zones and the possible molecular biological mechanisms

**DOI:** 10.3389/fonc.2023.1165732

**Published:** 2023-06-30

**Authors:** Xudong Yu, Ruijia Liu, Lianying Song, Wenfeng Gao, Xuyun Wang, Yaosheng Zhang

**Affiliations:** ^1^ Dongzhimen Hospital, Beijing University of Chinese Medicine, Beijing, China; ^2^ Beijing Tumor Minimally Invasive Medical Center of Integrated Traditional Chinese and Western Medicine, Dongzhimen Hospital, Beijing University of Chinese Medicine and Beijing Municipal Health Commission, Beijing, China; ^3^ Beijing Hospital of Traditional Chinese Medicine, Capital Medical University, Beijing, China

**Keywords:** prostate cancer, zones, clinical characters, heterogeneity, MRI

## Abstract

Since the theory of modern anatomical partitioning of the prostate was proposed, the differences in the incidence and pathological parameters of prostate cancer between the peripheral zone and transition zone have been gradually revealed. It suggests that there are differences in the pathogenic pathways and molecular biology of prostate cancer between different regions of origin. Over the past decade, advances in sequencing technologies have revealed more about molecules, genomes, and cell types specific to the peripheral and transitional zones. In recent years, the innovation of spatial imaging and multiple-parameter magnetic resonance imaging has provided new technical support for the zonal study of prostate cancer. In this work, we reviewed all the research results and the latest research progress in the study of prostate cancer in the past two decades. We summarized and proposed several vital issues and focused directions for understanding the differences between peripheral and transitional zones in prostate cancer.

## Introduction

According to the latest global cancer data in 2020 released by the International Agency for Research on Cancer (IARC) of the World Health Organization (WHO), prostate cancer (PCa) is still the second most common cancer in men worldwide. Also, PCa is one of the leading causes of cancer-related deaths in men worldwide (1). The American Cancer Society (ACS) recently released the 2023 cancer incidence and mortality report (2). The report shows that for men in 2023, prostate, bronchial lung, and colorectal cancers will account for 48% of all new cancers, with PCa accounting for 29% of all cancers in men. This proportion is a further increase from 2022 (2022: 27%) and represents the highest proportion of all new cancers in men (3). The continued rise in the incidence of PCa has caused great concern among cancer workers. The factors driving the progression of PCa during the formation of PCa cells are complex, and it is not sufficient to analyze only from the perspective of tumor cells (4). Since the McNeal zone method was proposed, the modern anatomical zoning theory of the prostate has been widely applied and demonstrated (5, 6). Clinical studies have reported that PCa mainly occurs in the peripheral zone (PZ), while only approximately 25% of PCa occurs in the transition zone (TZ) (7, 8). Several retrospective clinical studies have also found that PCa occurring in the PZ is more malignant compared to the TZ. PCa in the PZ tends to have the worse pathological stage and clinical outcomes (9–11). Meanwhile, PCa cells in the TZ are usually well differentiated, and their biological behavior and malignant potential differ from those in the PZ (12).

The differential pathogenetic features and biological behavior of PCa in the transitional and peripheral zones have attracted our attention. However, the biological mechanisms underlying this apparent difference between the different zones of the prostate are unknown. We hypothesized that this indicated the spatial distribution of PCa cells and tumor microenvironment heterogeneity. It may involve several factors, such as differences in matrix content and composition, gene fusions, androgenic mechanisms of action, and differences in genomic expression. Researchers have done some exploration from these perspectives. Therefore, the review summarized the different pathogenetic features and possible molecular biological mechanisms of PCa in the transitional and peripheral zones from the perspective of modern anatomical subdivisions of the prostate. We hope to provide a preliminary basis and research direction for further research.

## Division of the histological regions of prostate

### The historical evolution of the histological division of the prostate

**Figure 1:**
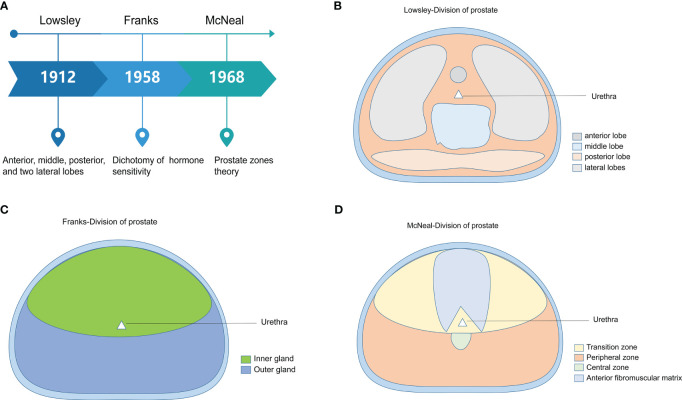
The historical evolution of the histological division of the prostate. **(A)** The three main methods of dividing the prostate are presented by time, researchers, and core perspectives. **(B)** Abridged general view of Lowsley’s five-lobe method of prostate dissection. **(C)** Abridged general view of Franks’ prostate hormone sensitivity dichotomy. **(D)** Abridged general view of McNeal’s prostate zones theory.

The anatomical division of the prostate can be traced back to the five anatomical classifications proposed by Lowsley in 1912 (13). Lowsley divided the prostate into anterior, middle, posterior, and lateral lobes. In addition, he held that the five lobes of the prostate had different origins during the embryonic period and gradually fused to form the main gland of the prostate after birth. However, in clinical practice, this five-point prostate anatomy approach differs from the actual clinical application. Franks proposed the dichotomy of prostate hormone sensitivity in 1958. He divided the prostate into inner and outer glands according to their sensitivity to sex hormones. The inner gland was the most common site of prostatic hyperplasia, and the outer gland was the most common site of PCa. This method is gradually being used less and less because it has yet to fully meet the needs of medical imaging development in recent years. McNeal proposed the zonal theory of the prostate from both histological and anatomical perspectives (5, 6). He divided the prostate body into glandular and non-glandular tissue. Glandular tissue is the major component. The glandular tissue area was divided into PZ, central zone (CZ), TZ, and a small part of the periurethral glandular tissue area. This method and theory are widely recognized and used in clinical practice. The prostate is the largest powerful organ of the male genital accessory gland. It comprises glandular and muscular tissues, of which muscular tissues account for approximately 30%, consisting of the detrusor muscle of the bladder neck that continues downward and the transverse muscle of the membranous urethra that extends upward (14). It is known as the anterior fibromuscular matrix area. Glandular tissue accounts for approximately 70%, which can be further divided into three main areas: TZ (approximately 5%–10% of the tissue), CZ (approximately 20%–25% of the tissue), and PZ (approximately 70% of the tissue) (6–8, 15). The historical evolution of the histological division of the prostate is shown in **Figure 1**.

### Histological features among normal prostate zones

Prostate tissue is composed of multiple cell types and involves multiple cell subsets. The glandular tissue region contains three epithelial cell types: luminal epithelial cells, basal cells, and neuroendocrine cells (16–18). Luminal epithelial cells, also known as secretory epithelial cells, are the primary cell type of prostate epithelial cells, accounting for approximately 60% of the total, and are considered functional secretory epithelium (19, 20). The secretory function of luminal epithelial cells is involved in semen production and is related to the quality of male sperm. Basal cells are located near the basement membrane and comprise approximately 40% of the cell types in the glandular tissue zone, and their primary role is maintaining duct integrity. The amount of neuroendocrine cells in the prostate tissue is minimal, less than 1%, and the specific function is unclear. The primary cell types in the stromal zone of the prostate are fibroblasts, myofibroblasts, endothelial cells, and immune cells (21). They are the main components of the prostate microenvironment and are also important factors affecting the occurrence and progression of PCa (22). Moreover, in addition to these cell types, recent studies have detected various rare prostate progenitor cells in the luminal epithelium and basal part of the prostate (23–25). These progenitor cells exhibit varying degrees of stem cell properties. Furthermore, some progenitor cells are the cells of origin of PCa (25, 26). We speculate that this may be a crucial factor affecting prostate tumor cells’ spatial distribution.

Different regions of the prostate have different histological features. The TZ had well-differentiated glands with irregularly sized epithelial cells arranged in a high columnar pattern and clear cytoplasm. The TZ had a denser stroma and lower nerve density than the PZ. In contrast, PZ is characterized by regular-sized epithelial cells and sparse stroma. The PZ was arranged in a corrugated pattern with a higher nerve density than the TZ and CZ. CZ is characterized histologically by large and irregular glandular vesicles. CZ has a larger nucleus, while the cell membrane is not apparent (7, 8, 14, 27). In addition, PZ and TZ were assigned different cell types. Epithelial cells of the prostate TZ were dominated by club cells, hillock cells, and PSCA+ epithelial cells. Fibroblasts dominated the stromal cells. In contrast, the epithelial cells of the PZ were dominated by luminal epithelial cells.

## Epidemiological characteristics of prostate cancer in PZ and TZ

Many pathomorphological observations have confirmed that PCa and prostatitis are more common in PZ. Benign prostatic hyperplasia (BPH) almost always occurs in the TZ. McNeal (28) pointed out that BPH does not manifest in PZ. CZ rarely involves carcinoma or hyperplasia. Very few tumors in the CZ of the prostate have been reported worldwide. There are such significant differences in the characteristics of prostate diseases in different regions, which has gradually attracted the attention of researchers. The pathological mechanism is still unclear.

### Tumor incidence and prostate zones

Over the half-century of clinical application of McNeal’s prostate zonation theory, more retrospective studies have revealed the epidemiological characteristics of PCa among different zones. The importance of the region of origin of PCa cells for clinical treatment and clinical outcome is also gradually being recognized. In 1988, McNeal et al. performed the pathological analysis of specimens from radical prostatectomy (RP). Approximately 70% of PCa were distributed in the PZ (28). Patrick et al. (11) analyzed the regional origin of the tumor in 7,051 patients after RP and individualized follow-up of the included patients. Their findings showed that PCa originating from PZ or CZ accounted for 80.54%, with high-grade disease (Gleason sum 4 + 3, 8, and 9 or greater) accounting for 42.33% of the cases. The percentage of PCa originating from TZ was 19.46%, with high-grade disease accounting for 19.90% of the cases. The study’s results by Patrick et al. are broadly consistent with some of the previous knowledge of PCa in TZ. Previous studies have shown that PCa originating from TZ had larger tumor volumes and higher PSA values (29–31). Instead, they had a lower Gleason score and a lower grade group (GG) (32–34). It implied that PCa with TZ is less aggressive and less likely to develop extraprostatic invasion (EPE) and seminal vesicle invasion (SVI) (35, 36). A study (37) statistically analyzed 607 tumor lesions from 180 specimens of whole RP in consecutive sections. A computer algorithm was used to map and summarize the regional distribution of these lesions. The study showed that 74% of the acquired lesions were in the PZ. Only 3 of the 180 PCa specimens were tumor lesions confined to the TZ. In addition, consistent with previous studies, lower Gleason scores and better tumor pathological stages were seen in a higher proportion of TZ. The other study (38) included 62 small-volume PCa samples for regional distribution statistics. The results showed that 79% of the tumors were in PZ, 16% in TZ, and 5% in CZ. This study also suggested that small-volume PCa was more likely to occur in the PZ and was usually multifocal and bilateral. Another study from Germany included 533 male patients diagnosed with PCa who underwent RP. This study combined the number of cancer foci, tumor volume, Gleason score, tumor area distribution, and pathological stage. The study showed that a positive biopsy (PBx) and a repeat PBx (rPBx) significantly differed regarding PCa spatial distribution. PCa diagnosed at either the first PBx or rPBx was predominantly located in the PZ of the prostate.

### Regional differences in incidence

It has been reported that the proportion of PCa patients distributed in TZ and PZ varies in different regions. One study included 370 cases after RP, including 159 cases from the United States and 211 from Japan. Approximately 35.3% of primary cancers in Japanese patients were TZ. However, this percentage is only 2.6% of patients in the United States. Another study from Japan included 638 patients with RP (10). PCa was found in 293 (46%) patients in the TZ group and 345 (54%) patients in the PZ group. Another study (39) performed pathological analysis on the prostates of 320 corpses with non-PCa causes of death, including 220 cases from Russia and 100 cases from Japan. The probability of PCa detection was 37.3% in Russian and 35.0% in Japanese men. The regional distribution of tumors showed that compared with the Russian population, the Japanese population had a higher proportion of PCa with TZ (25.9% vs. 20.7%). However, the difference in this proportion was not statistically significant, possibly due to the sample size.

From the available epidemiological data on the distribution of PCa zones, the incidence of PCa in TZ is higher in Asian populations than in Western countries. This difference reflects, to some extent, the natural trend of differences in incidence rates among regions. However, most of these epidemiologic data were based on retrospective single-institution studies of patients undergoing RP. Different research institutions need uniform standards for defining the region of origin of PCa. It may be an important cause of reporting bias in epidemiological studies. The data of PCa incidence differences in different regions reported in the epidemiological literature are summarized in **Table 1**.

**Table 1:** Clinical research data of spatial distribution of prostate cancer.

Author	Total cases (*n*)	Peripheral zone, *n* (%)	Transition zone, *n* (%)	Central zone, *n* (%)	Sample sources	Area, country	Data disclosure time	References
Takamatsu K et al.	638	345 (54%)	293 (46%)	NA	Radical prostatectomy	Asia, Japan	April 2019	(10)
Teloken PE et al.	7,051	5,679 (80.54%)^#^	1,372 (19.46%)	NA	Radical prostatectomy	Oceania, Australia	May 2017	(11)
Lee JJ et al.	1,354	1,124 (83%)	230 (17%)	NA	Radical prostatectomy	North America, United States	February 2015	(29)
King CR et al.	494	405 (82%)	89 (18%)	NA	Radical prostatectomy	North America, United States	November 2009	(40)
Chen ME et al.	180 (607 lesions)	74% of lesions	26% of lesions	NA	Radical prostatectomy	North America, United States	October 2000	(37)
Cheng L et al.	62	49 (79%)	10 (16%)	3 (5%)	Radical prostatectomy	North America, United States	August 2005	(38)
Cohen RJ et al.	726	655 (90.2%)	49 (6.7%)	22 (3.0%)	Radical prostatectomy	Oceania, Australia	May 2008	(41)

^#^The total number of peripheral zone and transition zone.

### Clinical prognosis and prostate zones

The primary zone of PCa is associated with clinical outcomes and multiple prognostic parameters. In most studies reporting clinical outcomes (biochemical recurrence-free survival, metastasis-free survival, or PCa-related death), patients with PCa diagnosed with TZ had better outcomes than those diagnosed with PZ (9–11, 42, 43). A study (40) retrospectively analyzed the surgical sample and clinical outcomes of 494 patients with PCa. Surgical specimens were sliced layer by layer to identify whether tumors originated from TZ or PZ. The 5-year biochemical relapse-free survival (BRFS) was better in 89 (18%) PCa patients with TZ than in those with PZ. Another study (44) performed precise clinical staging of 791 men treated only with RP (without radiation, chemotherapy, or androgen deprivation therapy). The results showed that the incidence of impalpable cancer in PCa patients with TZ was 2.5 times higher than in other zones. It suggests a better prognosis for PCa patients with TZ.

**Figure 2:**
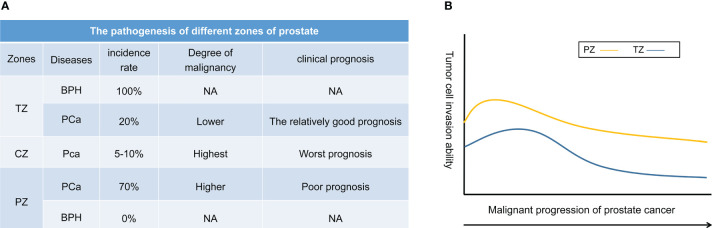
The illustration of prostate cancer zones with the combination of annotations of incidence and clinical prognosis. **(A)** The pathogenesis of different zones of prostate. **(B)** The relationship between malignant progression of prostate cancer and invasion ability of tumor cells predicted based on literature reports.

The study by Kimiharu et al. (10) also demonstrated a significantly better BRFS at years 3, 5, and 7 for PCa patients with TZ than for PCa with PZ (*p* = 0.012). It was confirmed by the study of Patrick et al. (11) that high-grade PCa originating from the TZ had a lower incidence of intraductal carcinoma, extraprostatic spread, seminal vesicle infiltration, lymph node involvement, and biochemical recurrence after RP. Another retrospective clinical study came to the same conclusion (29). The results of multivariate regression analysis showed that PCa originating from the TZ zone had a lower probability of seminal vesicle invasion, extracapsular extension, and lymphatic vessel invasion. PCa at TZ was independently associated with a reduced risk of tumor recurrence. However, a few studies found no significant correlation between the difference in tumor origin region and biochemical recurrence after RP (43, 45, 46). It may be related to the zone distribution and sample size. Therefore, there is considerable complexity regarding whether PCa primary zones have significantly different prognoses involving multivariate factors. In addition, very few tumors are reported to occur in the prostate in the CZ. According to the only reports in the literature, PCa located in CZ has the worst clinical outcome (41, 47). It may be related to the unique anatomical location of CZ. Because the CZ of the prostate contains the ejaculatory duct, and the seminal vesicle duct of the CZ opens at the prostatic urethra adjacent to the seminal vesicle, the specific anatomy of the CZ makes PCa occurring in this region more aggressive compared to the TZ and PZ. However, a higher level of evidence-based medicine still needs to verify this view. In conclusion, the overall clinical prognosis of tumors originating in different zones of the prostate varies widely. The clinical outcome of PCa localized in the TZ is the best prognosis of all zones. Trends in the incidence and malignant potential of different zones of the prostate are shown in **Figure 2**.

## Effect of prostate cancer on zone boundaries

### Physiological changes in the prostate zone boundaries

The volume of the prostate gland increases with age. It grows faster in volume in adolescence and old age, while in youth, its growth is relatively static. The growth of prostate volume during puberty is mainly due to androgen exposure, a physiological enlargement (48). After the age of 45, some men’s prostates begin to grow in size again at an accelerated rate. This phenomenon is pathological enlargement. The prostate volume increases further even after androgens decrease in older adults. There is no clear molecular biological evidence as to why BPH occurs in older men when androgen levels decline in the body. It may be related to various factors, including an imbalance of hormone levels in the body, high expression of dihydrotestosterone, and release of cytokines. The boundaries of the prostate zone are also in a dynamic process of change with physiological and pathological factors.

### Pathological changes in the prostatic zone

The frequency and magnitude of this dynamic change also increase with the incidence and age of prostate disease. The most typical manifestation is the change in the proportion of prostate TZ volume. When the prostate is mature, the proportion of TZ stabilizes at 5%–10% of the total prostate volume. With age or BPH, the TZ can expand to 30% of the total prostate volume by the time a man is 60 years old. In the process of this dynamic change, especially under the influence of significant prostate diseases, the original clear boundaries among zones will be distorted or even disrupted. For example, the autopsy study by Breslow et al. (49) proposed that the tumor distribution gradually spread from PZ to TZ as the PCa volume gradually increased. The original histological boundaries between zones may be destroyed in the tumor cell spreading to TZ.

### Value of MRI in identifying the region of origin of PCa

With the rapid development of medical imaging, especially the updated iteration of magnetic resonance imaging (MRI) technology, the accuracy of non-invasive examination of PCa has been dramatically improved. Now, we apply MPMRI to localize the region of origin of PCa better and measure the tumor volume. Applying MPMRI techniques makes it possible to design zonal studies of PCa. It will be valuable for understanding the diagnosis and prognosis of PCa in different zones. The primary imaging principle of MRI is based on the content of water and fat (50). The PZ and TZ of the prostate are different in this respect. Therefore, MRI is currently the optimal method for non-invasive examination of the prostate. Based on this principle, different MRI sequences have been optimized to interpret the different zones of the prostate. One study (51) re-read MRI in patients with biopsied PCa. There were 213 PCa lesions (68.9%) in the PZ area and 96 PCa lesions (31.1%) in the TZ area. Calculation of tumor cross-section size using MRI-related parameters showed that the tumor cross-section in the PZ zone was 14 mm and the TZ was 21 mm. Another study (52) evaluated the MPMRI features of PCa at PZ and TZ and compared them with whole histopathology. 3T-MPMRI showed that 76.7% of the lesions were located in PZ and 23.2% were located in TZ. In addition, the findings of MPMRI showed significant differences in tumor volume between the TZ and PZ regions. On 3T-MPMRI parameters, the median tumor volume in the TZ region was approximately 1.5 times that in the PZ. It is consistent with a retrospective study of postoperative samples of RP (29–31). TZ is associated with a larger tumor volume but a better pathological stage. In addition, although MPMRI is the best non-invasive examination method for PCa, there are still some difficulties in identifying the origin region of PCa. For example, cancerous lesions occurring in TZ and benign lesions such as BPH have similar features on MRI (53, 54). Therefore, assuming that prostate biopsy is abandoned altogether would result in a modest rate of misdiagnosis (55, 56). Nevertheless, for the diagnosis, localization, grading, and volume measurement of PZPCa, MPMRI is undoubtedly a fast and accurate method. In addition, magnetic resonance spectroscopy imaging (MRSI) can be used to assess the metabolic characteristics of the prostate (57). MRSI can provide the prostate’s metabolic information and help distinguish PCa from BPH (58). As we have described the unique lipid metabolism of the prostate in the previous section, it would be valuable to correlate the metabolic information of MRSI with metabolic abnormalities in PCa.

In conclusion, it is generally clear that PCa occurs more frequently in PZ (PZPCa). Meanwhile, approximately 10%–25% of PCa originated from TZ (TZPCa) (7, 59). Regarding regional distribution, available data show that the proportion of PCa with TZ is higher in Asia than Europe and the United States. Although these data may be subject to reporting bias, this may reflect a real trend. From the disease prognosis perspective, we know from the available data that PCa distributed in TZ has a lower Gleason score and better pathological stage. It suggests that PCa in TZ has a relatively better clinical outcome. However, the development and progression of PCa is a long and complex process. When the tumor cells spread beyond the original zone boundaries, identifying the origin of the PCa region became difficult. The disorder of the regional structure caused by significant prostate disease further increases the complexity of identifying the region of origin of PCa. In recent years, the wide application of multiple parameter magnetic resonance imaging (MPMRI) in the prostate provides a new method to solve this problem. The breakthrough of MPMRI technology has dramatically improved the accuracy of non-invasive detection of PCa (60, 61). At the same time, MPMRI makes it possible to identify the location of tumors and conduct PCa zone studies (62–64).

## Differences between PZPCa and TZPCa

The differences in the incidence characteristics of PCa among zones reflect biological differences. PCa occurring in different zones exhibit different histological features and molecular biological characteristics. For example, the matrix content and composition of PZ and TZ are different; There are differences in the frequency of TMPRSS2-ETS gene fusion and the expression of androgen receptors in different prostate zones (11, 65). Differences in biology among zones may be a potential driver of differences in the pathogenetic characteristics of PCa.

### Histological features between PZPCa and TZPCa

PZ is the high-incidence zone of PCa. The histologic features of PCa occurring in the PZ appear as infiltrating growths, small, round, or irregularly sized glands, but the boundaries of the glands are well defined. The glands is lined with cuboidal cells with amphiphilic cytoplasm and prominent nucleoli. It may present as cribriform and (or) glomeruloid glands with or without comedonecrosis. In addition, some also present as isolated cells or striated stroma. The histological features of PCa in TZ are small, intact tumor-like glands with round aggregates. The cytoplasm is dense and transparent. The round-like luminal epithelial cells show a nodular growth pattern similar to hyperplasia (66, 67). PCa occurring in TZ is often inevitably accompanied by BPH. As a result, the histological morphology of PCa is usually accompanied by hyperplastic histological changes. Some tumor cells may also grow invasively at the periphery of the hyperplastic nodule. MRI is also tricky to differentiate PCa from BPH in TZ. The nuclei of the PCa of TZ are continuously enlarged and hyperchromatic with prominent nucleoli. In contrast to PCa in PZ, glandular fusion and glomerular-like glands in TZ are rare (28, 66). PCa in CZ is extremely rare and less reported in the relevant literature. The histological features of PCa in CZ are similar to PZ morphology.

### Biological differences between PZPCa and TZPCa

A recent study (65) identified the cell types of PZ and TZ in the prostate tissues of older men by single-cell RNA-sequencing (scRNA-seq). The results showed that TZ aggregates more club and hillock cells than PZ. It was found that PZ contained more TFF3+ cells than TZ. However, the luminal cells of KLK3+ and IDH1 + 4 subgroups were more enriched in TZ. The expression of Notch pathway receptors (Notch1 and Notch2) and Notch signal transduction activity were significantly increased in club and hillock cells. Notch signaling is a driving force in regulating stem or progenitor cell biology in various tissues (68, 69). As mentioned, various rare progenitor cells have been detected in the prostate. Furthermore, they are associated with the origin of PCa (23–25, 70). It also suggests more excellent Notch pathway activity in the TZ zone of the prostate in older men.

In addition to different cell types and subsets, several genes are differentially expressed between different prostate zones. Studies have shown that differentially expressed genes (DEGs) between normal TZ and PZ persist in PCa from similar regions and are correlated with the Gleason score (71). A study (71) showed that the significant DGFs in prostate tissue PZ and TZ were as follows: BMP5, KIAA1210, TSPAN8, FOLH1B, TBX4, FOLH1, LAMA2, CPA3, FAM3B, CDH26, and TFPI. BMP5 was significantly upregulated in TZ but downregulated in PZ in normal prostate tissue.TSPAN8 and FOLH1B were significantly downregulated in TZ but upregulated in PZ in normal prostate tissue. The researchers evaluated these DGFs by establishing a prediction model. The results showed that the main reason for the differential expression of these genes was the difference in distribution zones. Regarding the above DGFs, it has been reported that BMP5 is a regulator of PCa progenitor cells and may be involved in bone metastasis of cancer cells (72, 73). FOLH1, also known as PSMA (prostate-specific membrane antigen), is involved in the occurrence and progression of PCa (74). In addition, several earlier studies have reported DGFs in different zones of PCa. For example, Sakai and colleagues’ (75) study found that the expression of Ki-67, MMP-2, and MMP-9 was significantly higher in PZPCa than in TZPCa. Another earlier study (76) compared the proliferation and apoptosis of tumor cells in TZ and PZ. The results showed that the apoptosis rate was similar between the two groups, but the proliferation rate of tumor cells in PZ was significantly higher than in TZ.

Moreover, p53 and bcl-2 were more frequently expressed in PZ than in TZ of PCa. Ki-67 has been proven to be an independent predictor of PCa death (77–79). High expression of Ki-67 and bcl-2 genes correlates with the invasive potential of tumor cells (80–82). It provides a possible molecular biological basis for the fact that tumor cells in the PZ are more susceptible to different prostatic spread than in the TZ. In addition, it was also found that the rate of PTEN loss was significantly higher in PZPCa than TZPCa (9). PTEN is a classic tumor suppressor gene that plays an essential role in PCa and many other cancers. The high deletion rate of PTEN in PZ is also an essential factor leading to the high incidence of PZPCa.

The differences in the molecular biology of PCa between TZ and PZ suggest different pathogenic pathways in different zones of PCa. Therefore, detection and analysis of molecular differences in different zones of the prostate can help reveal risk factors specific to different zones. Meanwhile, the region of origin of tumor cells should be an essential consideration in the study of diagnostic and prognostic biomarkers of PCa.

### Differential roles of androgen receptors in PZPCa and TZPCa

The therapeutic efficacy of androgen deprivation therapy (ADT) for PCa was first demonstrated by Huggins and Hodges back in 1941. Their study also revealed for the first time the critical role of androgens and their receptors in PCa (83). Most of the androgen that enters the prostate is produced in the testes, with a small amount coming from the adrenal glands, but it is generally thought to be less than 10%. The most common type of androgen in peripheral blood is testosterone. However, the dihydrotestosterone (DHT) ratio in prostate tissue is as high as 80%. Compared with testosterone, DHT has a stronger affinity with androgen receptors (AR) and has stronger biological effects (84). Testosterone primarily stimulates the normal physiological function of the testes and male muscle growth. After entering the prostate, testosterone is converted to DHT by the catalytic action of 5-α reductase. Alternatively, it is converted to estrogen by aromatase (85). DHT almost exclusively acts on the prostate, and the process of testosterone catalyzed by 5-α reductase is irreversible. Feneley et al. analyzed the spatial distribution of androgen receptors in BPH tissue. The results showed that PZ had a more significant AR enrichment than TZ. These findings suggest that PZ is more androgen-dependent than TZ under physiological and pathological conditions. Androgen and AR play a vital role in the occurrence and progression of PCa. It can be confirmed in ADT for high-risk PCa patients. AR signaling activity was maintained after androgen deprivation in studies of castration-resistant prostate cancer (CRPC) and metastatic castration-resistant prostate cancer (mCRPC). However, no studies have been reported on the spatial distribution of AR in PCa zones. Based on the limited studies available so far, this may be a biological basis for the differences in the characteristics of PCa pathogenesis between PZ and TZ.

Related to this is the TMPRSS2-ERG gene rearrangement or TMPRSS2-ERG gene fusion. TMPRSS2-ERG gene fusion has been detected in at least half of PCa patients (4, 86–88). It is the most common type of chromosomal rearrangement found in PCa. TMPRSS2 is a prostate-specific androgen regulatory gene. ERG is aberrantly expressed under the regulation of the androgen-responsive TMPRSS2 promoter (89, 90). PCa occurring in the PZ significantly overexpress ERG (9, 90). Chromosome immunoprecipitation analysis showed that ERG could bind to AR’s downstream target genes and block AR signal transmission in PCa cells by methylation silencing (91). It is consistent with the results of another study. Another study reported that the TMPRSS2-ERG fusion gene determines the fate of prostate cells by regulating chromatin interactions and is highly correlated with the phenotype of prostate tumor cells (92). The frequency of TMPRSS2-ERG fusion differs between TZ and PZ. The frequency of gene fusion was higher in the PZ region. However, TMPRSS2-ERG gene rearrangements are thought to be rare or absent in TZPCa (93–96). Whether the high frequency of gene fusions in the PZ is functionally linked to the enrichment of AR in the PZ needs to be further investigated.

### Differential metabolic profiles in PZPCa and TZPCa

Abnormalities in metabolism are one of the characteristics of oncological diseases. The rationale is that tumor cells strive to alter their original metabolic mechanisms during malignant progression to meet the energy requirements for its proliferation and invasion (97). Unlike conventional lipid metabolism, lipid metabolism in the prostate is unique. It is because the high accumulation of zinc elements in prostate tissue alters the pattern of the tricarboxylic acid cycle. This specific mechanism of lipid metabolism in the prostate is also regulated by AR signaling (98). A high-fat diet was a factor in the malignant progression of PCa in a mouse model (99, 100). Multiple studies have observed an abnormal increase in fat synthesis in PCa, coupled directly to glucose and glutamine metabolism, and associated with poor prognosis in PCa (101–103). Meanwhile, the molecular biological mechanism of lipid biosynthesis in PCa also involves the stimulating effect of androgen (104, 105). There needs to be more literature comparing fat metabolism among different zones of PCa. Enhanced lipid biosynthesis within the PZ compared to the TZ has been reported in the literature (59). The expression of genes associated with abnormal lipid metabolism in the PZ suggests that lipid-rich priming is associated with the development of PCa (106). Metabolic reprogramming is an important feature of oncologic disease. Unlike the abnormal glucose metabolism commonly seen in other tumors, glycolysis is often inhibited in PCa at the early stage of the disease (107, 108). However, abnormalities in lipid metabolism are the more prominent metabolic reprogramming of PCa. The differences in metabolic reprogramming among different zones of PCa need to be further studied.

## Discussion

The prostate is the most critical substantive organ in the accessory gonads of the male reproductive system. Modern medicine divides the prostate into three main zones: peripheral, central, and transitional. Since the theory of prostate partition was proposed, it has been widely used and demonstrated. The differences in the pathogenesis, imaging characteristics, histological characteristics, biological behavior, and malignant potential among different prostate regions have been gradually revealed. Many pathomorphological observations have confirmed that PCa and prostatitis are more common in PZ. Epidemiological studies have shown that BPH almost always occurs in the transition zone. However, CZ is rarely involved in cancer or hyperplasia, and cancer in CZ is rarely reported worldwide. The biological behavior and malignant potential of different prostate regions are also different. PCa located in PZ has higher malignant potential and worse clinical outcomes. The zone of origin of PCa cells is also increasingly recognized as necessary for clinical management and outcome. The primary site of PCa is associated with multiple prognostic parameters. For example, PCa originating from TZ has a lower probability of seminal vesicle invasion, extracapsular expansion, and lymphatic vessel invasion. RI is the best method for the non-invasive examination of the prostate. Different MR imaging sequences have been optimized to characterize different regions of the prostate. Prostate PZ and CZ are typically characterized using T2-weighted images, whereas TZ is typically characterized using diffusion-weighted imaging sequences. The marked variability in different zones of the prostate attracted our attention.

Androgen and AR play an essential role in the physiology and pathology of the prostate and are also the key molecules mediating the occurrence and development of PCa. The differential enrichment of AR in different prostate zones may be an important biological basis for the significant differences in the characteristics of the prostate between PZ and TZ. For a long time, the physiological and pathological relationship between androgens and their receptors in the prostate has been a research hotspot. In recent years, more and more studies have shown that estrogen and its receptors also play an essential role in normal or abnormal prostate biology. Estrogen affects target organs and tissues mainly by acting on the nucleus’ estrogen receptor (ER) (109). Classical ER is divided into two types, ERα and ERβ. Studies have shown that the proliferation of prostate stromal cells occurs through the action of estrogen on ERα on the stromal cells. ERβ is expressed in all stages of prostate development. ERβ plays a role in the differentiation of prostate epithelial cells and has an inhibitory effect on the proliferation of prostate cells. Studies have shown that the expression of ERβ in prostatic epithelial cells decreases in adult male patients, accompanied by prostatic hyperplasia and structural abnormalities (110). In summary, there is considerable evidence that estrogen and its receptor (ER) play essential roles in prostate growth and homeostasis. This effect is achieved by differential regulation of ERα in stromal cells and ERβ in epithelial cells. However, no studies have reported differences in the spatial distribution of ER in the prostate.

Heterogeneity in the spatial distribution of PCa has been widely reported. However, there are some studies that are limited to PCa itself. Ye et al. explored a novel approach to predict risk factors for tumorigenesis (111). They predicted a potential relationship between perfluorinated compounds and bladder cancer through TCGA combined with CTD. The results suggest that perfluorinated compounds can promote the progression of bladder cancer. The study method of Ye et al. provides more ideas for our research in PCa. Zhang T et al. predicted the possible carcinogenic effects of harmful substances on the prostate based on pharmacological techniques (112). Their results suggested that phthalates have an androgen-independent carcinogenic effect on PCa. The study by Zhang et al. expands our previous understanding of the biological mechanisms underlying the malignant progression of PCa. We need to comprehensively consider the pathogenesis of PCa from more directions, especially the formation factors of CRPC and mCRPC after ADT.

## Conclusions and prospects

It is important to explore the biological differences in different zones of PCa to reveal the origin of PCa cells. It will also be of great significance to explain the current drug resistance of PCa-related drugs, guide clinical treatment, and improve clinical efficacy. However, selecting “sufficiently standard” prostate tissue as the control group is a problem that needs attention in future research protocols. Paracancerous tissues are often selected as controls in studies of tumor heterogeneity. For the study of the prostate zone, this may ignore the potential cellular variation adjacent to cancer. It is difficult for researchers to obtain samples that match the diseased prostate tissue as a control. Because it involves the ethical issues of scientific research, this has been the main biasing factor in previous studies. However, the development of medical imaging, especially the innovation of MPMRI and MRSI technology, has brought new research ideas to solve this problem. Future studies can identify the region of origin of PCa with the help of sufficiently mature MPMRI and MRISI techniques. With MPMRI-guided prostate targeted biopsy, we will be able to target tissue samples from different zones of the prostate. Furthermore, more rigorous clinical and biological studies will be carried out. It is expected to accurately evaluate the relationship among lesions in different prostate zones and metabolism and prognosis.

In this work, we review all the research findings and recent advances in the study of PCa zones in the last two decades. We summarize and propose several key questions and focus directions for understanding the differences between PCa in PZ and TZ. Based on this differential pathogenesis and specific biological basis, we hope that the region of origin and current location of PCa can be included in future clinical diagnosis and treatment reports. It could provide more reliable clinical data for future retrospective and prospective studies. In addition, studies on the interzonal molecular biology of PCa still need to be improved. The results of high-quality clinical trials and basic research will be an important basis for whether the regional differences of PCa can be included in the risk classification criteria.

## Author contributions

XY and RL participated in the conception and design of the review. XY and RL drafted the manuscript. WG, LS, and YZ discussed and revised the manuscript. YZ and XW put forward some constructive suggestions. All authors contributed to the article and approved the submitted version.
